# Enhancing operative documentation of emergency laparotomy: a systematic review and development of a synoptic reporting template

**DOI:** 10.1186/s13017-023-00523-6

**Published:** 2023-11-30

**Authors:** Aiman Elamin, Emma Walker, Michael Sugrue, Syed Yousaf Khalid, Ian Stephens, Angus Lloyd

**Affiliations:** 1 Department of Surgery, Letterkenny University Hospital and Donegal Clinical Research Academy, Letterkenny, Ireland; 2 Department of Surgery, Beaumont Hospital, Dublin, Ireland

**Keywords:** Emergency laparotomy, Synoptic reporting, Operation notes, Patient safety, Digital transformation

## Abstract

**Introduction:**

Currently, operative reports are narrative and often handwritten, making interpretation difficult and potentially omitting key steps of the procedure. This study undertook a systematic review to determine the current availability of synoptic operative reporting and develop a synoptic operative record template for emergency laparotomy (EL).

**Methods:**

A PROSPERO registered study from January 1st, 2012, to December 31st, 2022, was conducted using PubMed, Scopus, and Web of Science databases in February 2023. Keywords: emergency laparotomy AND operation notes OR operative notes OR documentation OR report OR pro forma OR narrative OR synoptic OR digital OR audio-visual. Studies on paediatric or pregnant patients, systematic reviews, meta-analyses, case reports, editorial comments, and letters were excluded. A synoptic operative record was designed to include key standards in the documentation, as suggested by the Colleges of Surgeons.

**Results:**

The literature search yielded 4687 articles, and no relevant published articles were found. A detailed synoptic template was developed, which included 111 fields related to patient demographics, operative findings, interventions, and documentation of key variables associated with patient outcomes. 11 were text boxes, two were related to digital audio-visual uploads, and three facilitated the digital scoring/grading of findings.

**Conclusion:**

This systematic review identified a limited number of publications reporting synoptic operative reporting, and none related to emergency laparotomy. This novel operative template provides a platform for clear documentation of the surgery performed during emergency laparotomy, potentially facilitating data analysis, resident training, and research, in turn leading to a better understanding of patient outcomes.

## Introduction

The global burden of surgery and challenges in the delivery of emergency general surgery (EGS) are becoming increasingly important in the delivery of healthcare. Emergency general surgery accounts for approximately 10% of hospital admissions, with an average 770/100,000 of population globally. 20% of these patients undergo surgery, of which laparotomy is the cornerstone of emergency surgery [1, 2].

Emergency operations are associated with significant morbidity. Patients undergoing EGS procedures are up to eight times more likely to die than those undergoing the same procedure electively [3, 4]. Emergency general surgery lacks a robust quality and performance improvement process, data collection tools, and standards to guide the organization of programs based on the optimization of resources, processes, and measured outcomes [5]. Recent innovations by the American College of Surgeons to advance standards follow the initiatives of the World Society of Emergency Surgery working with the Donegal Clinical Research Academy on performance indicators in emergency surgery [6–8].

A clear, team-wide understanding of the operative procedure and postoperative plan is vital for improving the outcomes [9]. Operative records are fundamental to the documentation process, highlighting the indications, incision, procedure, closure of the abdomen, and postoperative instructions. Several institutions including the Royal College of Surgeons (RCS Eng.) and the American College of Surgeons (ACS), have advocated various guidelines for documenting operative records [10, 11].

For over a century, there have been many attempts to improve record-keeping owing to the recognition of problems with handwritten and narrative operative records. Over 50 years ago, it was suggested that operation records should be typed and potentially include cinematography and photographs to enhance the documentation process [12, 13]; however, these have not been widely adopted, and current operation records are universally hand-written. Few opt for a synoptic digital system despite the remarkable global transformation in digital technology.

Synoptic reporting templates provide a clear, prompt guide for surgical record keeping, which are comprehensive, inclusive, and offer a staged approach to procedures. In addition, they can provide prompts to facilitate scoring and grading systems, the inclusion of audio-visual aids, and reduce errors of omission. However, they are time-consuming and may take longer to complete than a handwritten or dictated report [14]. The increased workload and need for change have resulted in resistance to broader adoption [15].

Recent guidelines from the World Society of Emergency Surgery (WSES) looking at more effective closure of laparotomy in emergency settings (ECLAPTE) have identified several key pivotal recommendations for laparotomy closure but do not address operative record documentation [16].

Synoptic operative records provide a potential opportunity for the inclusion of digital hyperlinked educational grading systems and technical tips. Synoptic operative records provide a database for research on patient surgery and its impact on the outcomes. Synoptic operative records enhance residents’ training by prompting the identification of key stages of procedures that must be completed and documented [17]. Currently, however, few synoptic operative reporting systems are in place [11, 18]; therefore, this study undertook a systematic review of the literature to determine the current availability of synoptic operative reporting and develop a synoptic template for the operative record documenting emergency laparotomy.

## Methods

### Search strategy

A search was conducted using the PubMed version of Medline, Scopus, and Web of Science electronic databases for all relevant articles published in February 2023. The keywords used included emergency laparotomy AND operation notes OR operative notes OR documentation OR report OR pro forma OR narrative OR synoptic OR digital OR audio-visual. The search included all articles published from 1 January 2012 to 31 December 2022.

### Inclusion and exclusion criteria

**Fig. 1:**
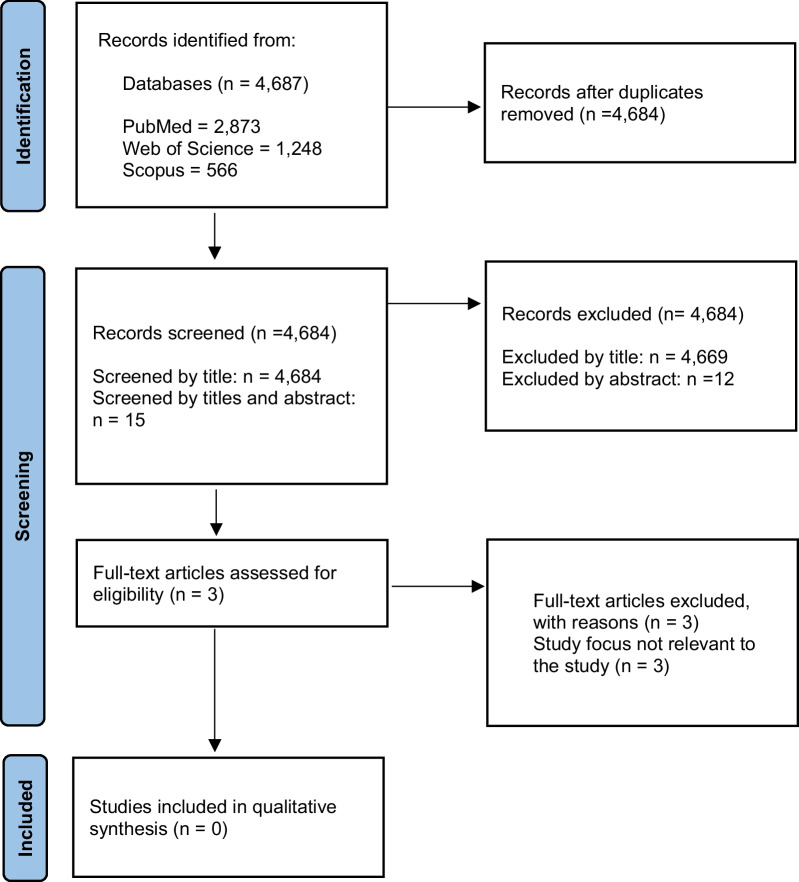
PRISMA flow diagram: identification, review and selection of articles included in this systematic review

The methods and study inclusion criteria were specified in advance, and the protocol was registered with the International Prospective Register of Systematic Reviews (PROSPERO) database registration number CRD42023396649 [19]. The Preferred Reporting Items for Systematic Reviews and Meta-Analyses (PRISMA) guidelines were followed in this systematic review (Fig. 1) [20]. Articles written in English and full-text reporting studies on the operative documentation of EL were eligible for inclusion. The study did not include paediatric (< 16 years of age) patients or studies on pregnancy, as well as systematic reviews, meta-analyses, case reports, editorial comments, and letters. Microsoft Excel® was used to import citations, and duplicates were removed. The references of the reviewed studies were examined for papers not identified by the initial search strategy.

### Study selection and data extraction

Upon completion of the search, screening for inclusion was performed, initially by title and then abstract, followed by a full-text review. Two reviewers (AE and EW) assessed eligibility. A consensus was used to resolve disagreements; however, a third reviewer (IS) was involved when no agreement could be reached.

## Results

The literature search produced 4687 articles. 4684 publications were assessed after duplicates were eliminated: 4669 were excluded based on their titles, 12 more were excluded based on their abstracts, and 3 were eliminated after a full-text review. No articles relevant to this study were found during the search (Fig. 1).

**Fig. 2:**
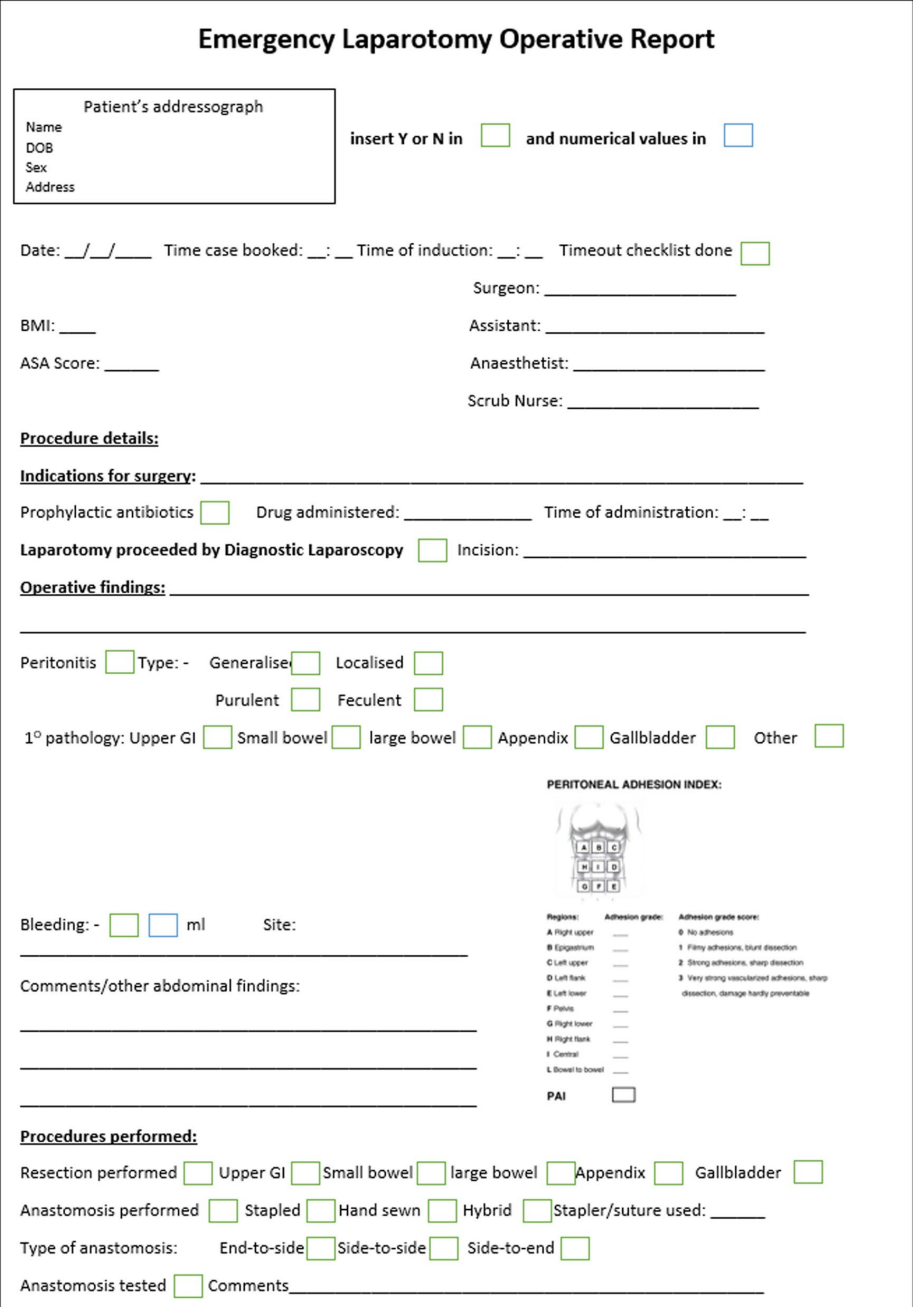

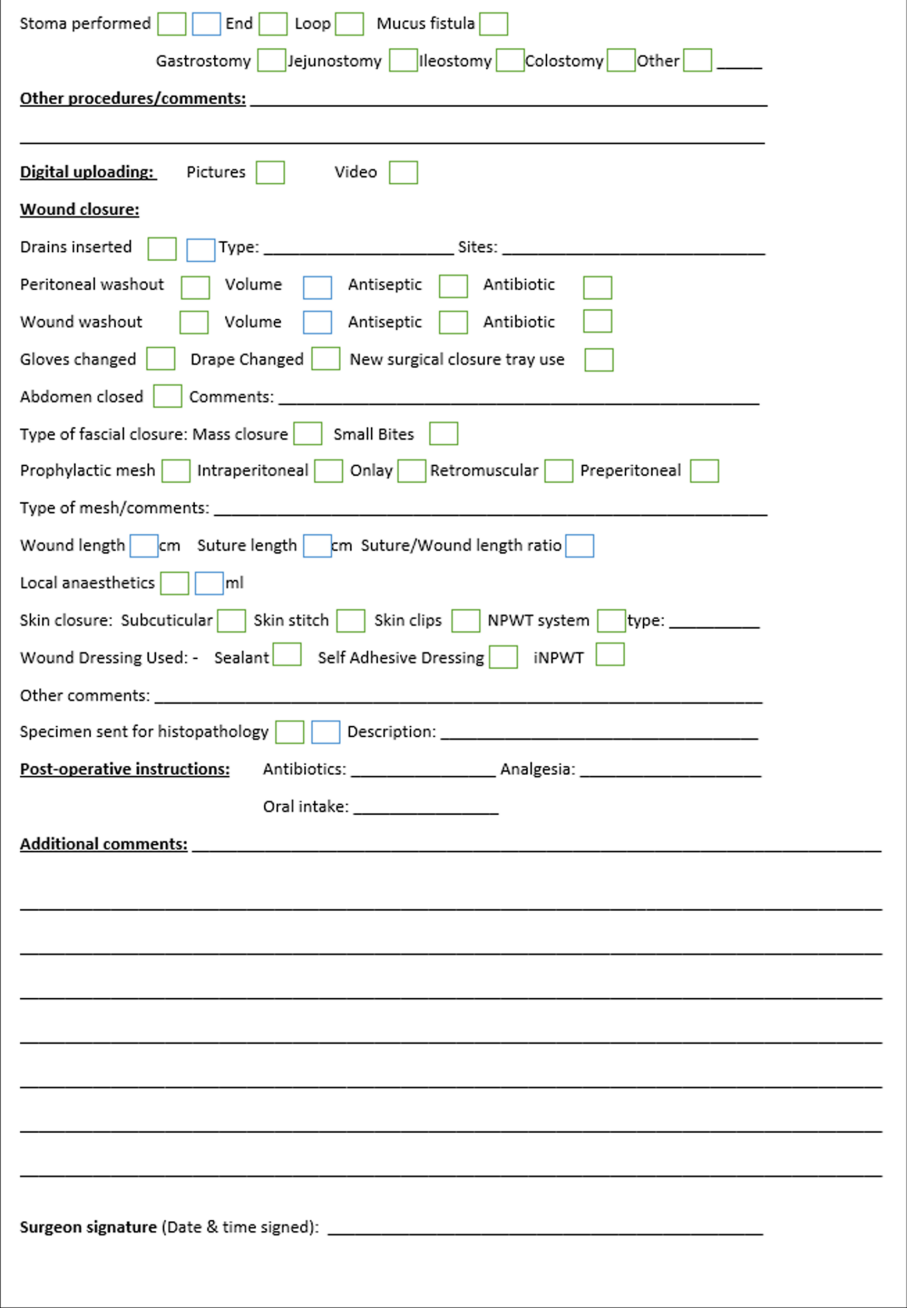
Proposed emergency laparotomy operative template

The synoptic report developed, as shown in Fig. 2, includes key fields suggested by the Royal College of Surgeons of England [10] as fundamental requirements for an operative record including the booking procedure and comorbidity status. The indications for surgery, use, and type of antibiotics are recorded. The nature and cause of peritonitis are documented. The digital version of the report will allow automatic digital scoring of intraperitoneal adhesions whilst also allowing for upload of both pictures and videos depending on the hospital IT connectivity and medical record status. In the absence of functioning electronic medical records, the operative records can be handwritten and filed in the patients’ medical records. The operative report also contains detailed documentation of both anastomotic technique and abdominal wall closure. Additional comments can also be provided. A total of 111 fields related to patient demographics, operative findings, intervention, and documentation of key variables associated with patient outcomes were developed [21].

## Discussion

This systematic review revealed a lack of publications reporting the design and use of synoptic operative records for emergency laparotomies. Emergency laparotomy is a life-saving procedure that should be properly and informatively documented to serve the best interests of patients and surgeons.

Hospital documentation is a legal requirement that all doctors must meet [22]. Operative records are paramount for exchanging patient information between surgeons and other professionals. Service and quality of care can be improved by structuring and organizing operative documentation to maintain the highest standards of continuity of information and care.

Historically and in most institutions, operative records were hand-written narrative records. Operative record documentation is rarely taught to juniors. In a multicentre study of laparoscopic cholecystectomy operation records in nine hospitals in the Netherlands, Wauben et al. [23] found that 30% of the operative records did not comply with the Dutch guidelines. They recommended including operative record writing in the surgical training program. Melton et al. [24] conducted a national survey to evaluate surgical training programs including operative report documentation and usage of synoptic or template report, and while finding that most program directors considered operative documentation training an educational priority, this was not widely adopted in most surgical specialties. In 2017, an audit of general surgery and urology handwritten operative records was conducted by Nzenza et al. [25] using the Royal College of Surgeons of England guidelines, which found significant deficiencies. They concluded that there was a noticeable lack of training in how to write operative records. Nyamulani and Mulwafu [26] conducted a prospective review of the quality of operative records, identifying critical deficiencies in operative documentation and concluding that educating doctors using a proforma would improve their completeness. Apramian et al. [17] conducted a qualitative analysis of operative records of patients undergoing tonsillectomy and found that operative records, while essential for documentation, can be used as a learning and assessment tool.

Standardized synoptic reporting systems can potentially enhance the feasibility and reproducibility of surgical research. Standardizing surgical operative records may prompt residents to avoid omission in a particular operation and may result in reduced complications. An example would be the requirement to document the suture-to-wound ratio, which will help reduce the incisional hernia rate and wound dehiscence. The medicolegal aspect of indecipherable operative records can be addressed by the introduction of synoptic operative reporting fields, especially if digitally completed [27].

A consensus on the data elements and fields to be completed for emergency laparotomy was reached following departmental discussion. This consensus has been successfully achieved in other areas, such as lung cancer surgery, when Schneider et al. [28] used an online survey to create a consensus and optimized surgical documentation and utility of information. This has been achieved in colorectal surgery with more reliable and accurate documentation of the rectal cancer checklist [29]. Kanters et al. [30] conducted a multi-centre study in 2018 assessing the completeness of operative records for rectal cancer surgery to check whether recommendations of the National Accreditation Program for Rectal Cancer were followed. In a study of ten hospitals, rectal cancer operative records were reviewed, and it was concluded that synoptic reporting of rectal cancer surgery is associated with better-quality operative records.

Thomson et al. introduced an operation reporting pro-forma for laparoscopic cholecystectomy in a tertiary centre in the UK in 2016, followed by an audit of 128 operative records. They concluded that implementing procedure-specific pro forma leads to more accurate and robust medico-legal documentation [31]. In 2019, a comparative review of synoptic operative reporting versus narrative operative records focusing on both user-friendliness and completeness of the historical narrative report to the synoptic operative report concluded that there was a higher completion and accuracy rate combined with a lower completion time when using the synoptic operative record compared to the traditional narrative record. Similar findings supporting the advantages of synoptic reporting have been found in other studies [32–34]. They also concluded that there is potential for better completion and accuracy rates when using synoptic operative reporting systems in a hybrid approach of narrative and synoptic methods which will lead to higher satisfaction among surgeons and other healthcare professionals [32]. In 2019, a systematic review and meta-analysis conducted by Stogryn et al. found that synoptic operative reports outperformed narrative reports [33]. In 2020, Robertson and Vergis [34] conducted a prospective comparative study to evaluate preoperative and intraoperative quality of care documentation in traditionally dictated reports. They compared it to synoptic reports for rectal cancer surgery and concluded that the synoptic reporting method resulted in more accurate documentation compared to traditional dictated reporting methods. In 2021, St John et al. [35] conducted a prospective study to evaluate the consent process and associated documentation in breast and general surgery and concluded that there were high error rates and omissions associated with handwritten forms compared to a standard template. Dyke et al. [36] conducted a study to evaluate the legibility, accuracy, and completion of the consenting process and to compare paper consent forms to digital forms. They included 223 patients who consented by using either paper consent forms or digital forms. They found that there were one or more errors associated with paper consent forms compared to zero errors associated with digital ones; therefore, they concluded that using concentric digital consent platforms can improve the quality of the consenting process by reducing errors and is associated with better patients’ decision-making experience.

There are several limitations to the current study in that the pro forma, although developed by consensus, has not been evaluated to assess its long-term acceptance. In addition, the digital pathway to allow the uploading of digital images, videos, and automated scoring systems has not been finalized. However, it has not been subjected to cost analysis.

## Conclusion

This systematic review revealed the absence of scientific publications on synoptic operative record documentation of emergency laparotomies, despite strong evidence that standardization of operative records with training is associated with higher accuracy and completeness of operative records. The proposed new operative template will improve the documentation of emergency laparotomies, which could lead to better outcomes, training, and research.

## Data Availability

Data sharing is not applicable to this article, as no datasets were generated or analyzed during the current study.
